# Chinese Americans’ Information Sources on, Preferred Types of, and Satisfaction with COVID-19 Vaccination

**DOI:** 10.3390/vaccines11121823

**Published:** 2023-12-06

**Authors:** Ming Li, Zuojin Yu, Bo Kyum Yang, Xuewei Chen, Gary L. Kreps

**Affiliations:** 1 Department of Health Sciences, Towson University, Towson, MD 21252, USA; zyu@towson.edu (Z.Y.); byang@towson.edu (B.K.Y.); 2 School of Community Health Sciences, Counseling and Counseling Psychology, Oklahoma State University, Stillwater, OK 74078, USA; xuewei.chen@okstate.edu; 3 Center for Health & Risk Communication, George Mason University, Fairfax, VA 22030, USA; gkreps@gmu.edu

**Keywords:** Chinese Americans, COVID-19 vaccine, satisfaction, information sources, preferred types

## Abstract

According to the Centers for Disease Control and Prevention (CDC), about 87% of Asian Americans had received at least one dose of the COVID-19 vaccine as of July 2023. The purpose of this study is to identify the sources of information, preferred vaccine types, and levels of satisfaction related to COVID-19 vaccination among Chinese Americans, the largest subgroup of Asian immigrants living in the U.S. Our survey data were collected from 241 Chinese American early adopters of the COVID-19 vaccine, who completed at least one dose of the COVID-19 vaccine in June 2021. Our results indicated that their major information sources regarding COVID-19 vaccination included health officials and authorities, local news, family/friends/co-workers, social media platforms, and healthcare professionals. More than half of the participants expressed a preference for the Pfizer-BioNTech (New York, U.S.) vaccine based on the primary considerations of safety, efficacy, credibility of the developer, and availability. A majority of the participants felt satisfied with their experience of receiving the COVID-19 vaccination. Participants with higher levels of self-efficacy and subjective norms related to receiving the COVID-19 vaccine were more likely to express satisfaction with the vaccination. These findings provide valuable insights into Chinese Americans’ information sources, vaccine preferences, and satisfaction levels regarding COVID-19 vaccination. This knowledge can help guide future vaccination interventions and campaigns.

## 1. Introduction

As of May 2023, according to the Centers for Disease Control and Prevention (CDC) COVID-19 Vaccine Tracker, 81.4% of the U.S. population received at least one dose of the COVID-19 vaccine, 69.5% completed the primary vaccine series, and 17.0% received an updated (bivalent) booster vaccine dose [1]. As of July 2023, 87% of Asian Americans had received at least one dose of the COVID-19 vaccine [2]. Up to November 2023, the implementation of the COVID-19 vaccination and its boosters was ongoing to help enhance immunity against COVID-19 infection and to improve health outcomes for those who became infected with the virus. This achievement marks a historical moment in the rapid development, distribution, and administration of a new vaccine.

Throughout the pandemic, people accessed health information from various channels to receive information about the COVID-19 vaccine. A study conducted in the U.S. documented that individuals who received information via traditional media sources such as national television, national newspapers, and local newspapers were most likely to receive the COVID-19 vaccine [3]. Another study reported that individuals with high trust in mainstream sources in the U.S. (e.g., the CDC, state health departments, academic or research institutions, and individual healthcare providers) used these sources with the intention of encouraging their family members to receive COVID-19 vaccinations [4]. However, individuals who had high levels of trust in politically conservative sources used these sources to discourage friends from getting vaccinated [4]. Different population groups may receive information from varied sources regarding COVID-19 and vaccinations. According to the U.S. Census Bureau, there are about 4,521,970 Chinese people living in the U.S. as of 2022 [5], and Chinese Americans (CAs) make up one of the largest immigrant groups in the U.S. [6], with approximately 78% of the CAs who are 18 years old or above born outside of the U.S. [7,8]. CAs’ sources of information regarding the COVID-19 vaccine may be different from other populations in the U.S. given their different language and cultural background. For example, although most of the CAs received a relatively high level of education, only 39% of Chinese immigrants in the U.S. speak English fluently, which may limit their access to health information sources provided only in English [7,8]. Despite the previous literature that documented CAs’ sources of general health information [9], conclusions about how CAs access the information regarding COVID-19 vaccination are still unclear.

The COVID-19 vaccines authorized by the U.S. Food and Drug Administration (FDA) include the Pfizer-BioNTech (New York, U.S.) vaccine (an mRNA vaccine), Moderna (Cambridge, U.S.) COVID-19 vaccine (an mRNA vaccine), Novavax (Gaithersburg, U.S.) COVID-19 vaccine (a protein subunit vaccine), and Johnson & Johnson’s Janssen (New Brunswick, U.S.) COVID-19 vaccine (a viral vector vaccine that is no longer available for use in the United States as of 6 May 2023) [10]. One study investigating COVID-19 vaccine acceptance in China found that 32.5% of participants preferred domestic vaccines (vaccines made by Chinese manufacturers), while only 3.3% of the research participants preferred imported vaccines (vaccines developed by manufacturers outside of China, e.g., Pfizer and Moderna) [10]. Failing to receive their preferred type of COVID-19 vaccine may influence CAs’ overall vaccine satisfaction.

Satisfaction, in the context of vaccine users, refers to their personal perceptions of how happy or satisfied they are with the vaccine service they received [11]. This is measured by the extent to which the participants are satisfied with their experiences of getting vaccinated. Having a high level of vaccine satisfaction may encourage the uptake of an additional dose of the COVID-19 vaccine or other types of vaccines (e.g., HPV vaccine and flu vaccine). For instance, our previous research has demonstrated that CAs who expressed satisfaction with their prior COVID-19 vaccination experiences were more willing to receive additional hypothetical annual COVID-19 vaccine doses in the future [12]. Other studies found that self-efficacy and influences from people who are important to them are significantly related to vaccine satisfaction among the US general population [13]. Although our previous study found influence of satisfaction with previous vaccinations on individuals’ decision on future vaccination, the evidence on what drives those unique perspectives toward the COVID-19 vaccine among this specific population is still lacking, requiring further investigation.

In the present study, we analyzed the data collected from a research project designed to examine CAs’ perspectives toward and experiences with COVID-19 vaccination [12]. In our previous article, we found that satisfaction level, knowledge, and perceived susceptibility were significantly associated with CAs’ willingness to receive the annual COVID-19 vaccine in the future [12]. In the current study, we extended our examination to uncover the factors associated with early adopters’ satisfaction with COVID-19 vaccination using the same data collected for our previous research. Specifically, the purpose of the present study is to seek answers to the following questions. (1) What were CAs’ primary information sources regarding COVID-19 vaccines? (2) Did CAs have a preferred type of COVID-19 vaccine? (3) What are the factors influencing CAs’ satisfaction with COVID-19 vaccination?

## 2. Materials and Methods

The data used in the present study were collected as part of a research project designed to assess CAs’ attitudes, knowledge, intentions, and behaviors regarding COVID-19 vaccination and boosters. The survey was developed based on the past literature, health behavior theories (i.e., the Health Belief Model and the Theory of Planned Behavior), and the Expectation Confirmation Model (ECM) [14]. Detailed information regarding survey development and participant recruitment was described in our previous publication [12]. The study protocol was approved by the Towson University Institutional Review Board.

### 2.1. Study Population and Process

The eligible participants in this study included CAs living in the U.S. aged 18 years or older who had received at least one shot of a COVID-19 vaccine. Online data collection lasted two weeks (5 June 2021 to 13 June 2021), starting one month after the Secretary of Health and Human Services issued a directive to expand COVID-19 vaccine eligibility to all people in the U.S. instead of just for high-priority groups [15]. Participants were recruited from various CA communities, organizations, and groups of social media frequently used by CAs. Information sheets were provided, and participant consent was obtained before filling out the surveys. The survey was made available in two different language versions: English and Chinese. Participants could choose their preferred language to fill out the survey. Each participant received a $10 electronic gift card as an incentive after completing the survey.

### 2.2. Measures

We asked participants to choose their primary sources of information regarding the COVID-19 vaccine from a list of nine information sources (i.e., healthcare professionals, health officials, local news, family/friends/co-workers, social media, celebrities/public figures, religious leaders, political leaders, and other). These sources were selected based on findings from the previous literature [16,17]. Participants could pick more than one information source option.

Self-efficacy for receiving the COVID-19 vaccine refers to beliefs about the individual’s own ability to receive the COVID-19 vaccine to achieve the desired outcome of preventing serious illness and death from the COVID-19 virus. This construct was measured with 3 items adapted from previous studies (e.g., “I feel confident in making an appointment to receive a COVID-19 vaccine.” 1 = Strongly disagree; 5 = Strongly agree) [18]. Cronbach’s α was 0.824.

Subjective norms for getting the COVID-19 vaccine refer to individuals’ beliefs about whether peers and people of importance think he/she/they should get vaccinated. We used 3 items adapted from previous studies to measure this construct (e.g., “Most people who are like me will get vaccinated for COVID-19.” 1 = Strongly disagree; 5 = Strongly agree) [18]. Cronbach’s α was 0.858.

Satisfaction with COVID-19 vaccination was measured with one item: “To what extent are you satisfied with your experience of getting the COVID-19 vaccine? 1 = not at all satisfied; 5 = extremely satisfied.”

Failure to receive the preferred type of COVID-19 vaccine in previous shot(s) was categorized as “yes” vs. “no” based on participants’ responses to two questions: (1) What is the manufacturer/developer of the COVID-19 vaccine that you actually received or will potentially receive? and (2) What is your preferred manufacturer/developer of the COVID-19 vaccine? Detailed information regarding the measurement of this variable is described in our previous publication [12].

In the present study, acculturation was measured using two subscales from the Asian American Multidimensional Acculturation Scale (AAMAS) [19]: Culture of Origin (AAMAS–CO) and European American Culture (AAMAS–EA) [20]. For each item, respondents indicated on a 6-point scale (1 = not very well; 6 = very well). The AAMAS subscale scores were based on the average rating (from 1 to 6) for each scale across the 15 items. For the AAMAS–CO and the AAMAS–EA, Cronbach’s α were 0.881 and 0.925, respectively.

In addition, sociodemographic information including participant’s age, gender, birthplace, marital status, educational level, religion, employment, and annual household income was collected in the survey.

### 2.3. Data Analysis

First, we assessed the frequencies, percentages, means, and standard deviations of each variable. Bivariate analyses were performed to examine the relationship between each predictor and satisfaction. Bivariate analyses were performed to examine the relationship between outcome variables (information sources, preferred type of vaccination, self-efficacy, and satisfaction) and predictors. Specifically, we used bivariate correlations to examine the relationships between continuous outcome variables (i.e., self-efficacy) and predictors; modified Poisson regressions were used to examine the relationship between categorical outcome variables (i.e., information sources, preferred type of vaccination, and satisfaction) and predictors [12,21,22]. For satisfaction, significant predictors from bivariate analyses (*p* < 0.05) were included in a multivariable modified Poisson regression model to assess factors associated with CAs’ satisfaction with the COVID-19 vaccination. All statistical analyses were conducted using STATA Version 17.0 with *p* < 0.05 as the significance threshold.

## 3. Results

### 3.1. Demographic Characteristics

A total of 241 participants completed this survey. Table 1 shows the characteristics of our sample. More than half of the participants (n = 138, 57.3%) chose English as the language to fill out the survey, with the remaining participants (n = 103, 42.7%) choosing the Chinese version of the survey.

### 3.2. Chinese Americans’ Information Sources Regarding COVID-19 Vaccine

**Figure 1:**
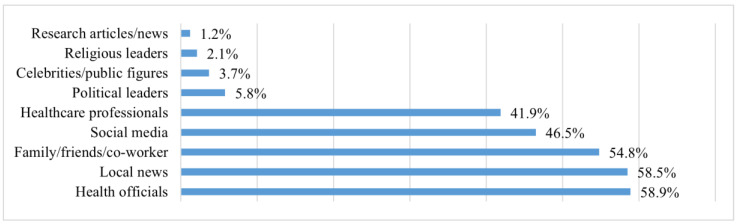
COVID-19 vaccination information sources.

As summarized in Figure 1, the five major information sources listed by the frequency from highest to lowest included (1) health officials and authorities (e.g., Center for Disease Control and Prevention, Department of Health, National Institute of Health, and World Health Organization) (n = 142, 58.9%), (2) local news (n = 141, 58.5%), (3) family/friends/co-workers (n = 132, 54.8%), (4) social media (n = 112, 46.5%), and (5) health professionals (e.g., doctors, nurses, and pharmacists) (n = 101, 41.9%).

Participants who were employed (adjusted PR = 1.403, CI: 1.101, 1.1786) and scored higher on AAMAS–CO (adjusted PR = 1.284, CI: 1.033, 1.597) were more likely to receive COVID-19 vaccine information from health officials and authorities. Participants who were employed (adjusted PR = 1.301, CI: 1.027, 1.649) were more likely to receive information from local news. Receiving lower scores in AAMAS–EA (adjusted PR = 0.852, CI: 0.765, 0.949) were associated with receiving information from family/friends/co-workers. Being employed (adjusted PR = 1.493, CI: 1.095, 2.035) and without religious affiliations (adjusted PR = 0.714, CI: 0.533, 0.955) were associated with receiving information from social media. Participants who received higher level education (adjusted PR = 1.257, CI: 1.033, 1.531), were born in the U.S. (adjusted PR = 1.528, CI: 1.012, 2.307), and received higher AAMAS–EA scores (adjusted PR = 1.229, CI: 1.0699, 1.412) were more likely to receive COVID-19 vaccine information from healthcare professionals.

### 3.3. Chinese Americans’ Preferred Type of COVID-19 Vaccine

Participants’ preferred type of COVID-19 vaccine listed by the frequency from highest to lowest included Pfizer-BioNTech (n = 152, 63.1%), Moderna (n = 33, 13.7%), Johnson & Johnson’s Janssen (n = 8, 3.3%), Novavax (n = 7, 2.9%), and either Pfizer-BioNTech or Moderna (n = 1, 0.4%). The rest participants (n = 40, 16.6%) reported no preference on the manufacturer of COVID-19 vaccine. The primary considerations of the preference regarding the manufacturer/developer of the vaccine included: safety (including side-effects) (n = 158, 65.6%), efficacy (n = 151, 62.7%), credibility of the developer (n = 55, 22.8%), and availability (n = 36, 14.9%).

Regarding the manufacturer/developer of the COVID-19 vaccine received, the majority of participants received Pfizer-BioNTech (n = 164, 68.1%). Over a quarter of participants received Moderna (n = 64, 26.6%). Nine participants reported receiving Johnson & Johnson’s Janssen (3.7%), one reported receiving Kexing (Jinan, China) (4.1%), and three reported “don’t know”. The majority of the participants received the type of COVID-19 vaccine they preferred (n = 202, 83.8%), while the rest of the participants (n = 39, 16.2%) failed to receive the type they originally preferred.

### 3.4. Chinese Americans’ Satisfaction Regarding COVID-19 Vaccine

The vast majority of participants in our sample (83.0%) expressed satisfaction with their prior experience with COVID-19 vaccination. Bivariate analyses showed that satisfaction was positively associated with self-efficacy and subjective norms regarding the COVID-19 vaccine. As shown in Table 2, when controlling by demographic factors, participants with higher levels of self-efficacy toward receiving the COVID-19 vaccine (adjusted PR = 1.158, CI: 1.012, 1.326) and a higher level of subjective norms toward vaccination (adjusted PR = 1.172, CI: 1.010, 1.360) were more likely to report satisfaction with vaccination. The participants who chose the Chinese language to answer the survey (adjusted PR= 0.795, CI: 0.681, 0.930) and participants who failed to receive the preferred type of vaccine (adjusted PR= 1.172, CI: 1.010, 1.360) were less likely to report satisfaction with vaccination.

## 4. Discussion

We analyzed the survey data collected from the previous research examining the factors associated with receiving a hypothetical annual dose of COVID-19 vaccine to extend our investigation to CA early vaccine adopters’ vaccination information sources, preferred types of COVID-19 vaccines, and factors associated with their satisfaction regarding COVID-19 vaccination. In our prior research involving 241 CA participants, we found that most respondents reported a positive experience with the previous vaccination. Additionally, participants with positive vaccination experience, with accurate knowledge regarding COVID-19 vaccines, and higher perceived risk of COVID-19 infection were associated with willingness to receive a hypothetical annual dose of COVID-19 vaccines. Our recent investigation contributes valuable new insights into the information-seeking behavior of this population regarding the COVID-19 vaccine. The primary sources of information we identified included health officials, local news, personal networks (family, friends, and co-workers), social media, and healthcare professionals. In addition, we found that CAs receiving the preferred type of vaccine, with higher levels of self-efficacy, and with higher levels of subjective norms toward vaccination reported higher levels of vaccine satisfaction.

Unlike people in China who had a high preference for domestic (Chinese) vaccines, few CA participants in our survey preferred vaccines from Chinese manufacturers, even though such vaccines were listed as options in the multiple-choice question asking for participants’ preferred manufacturer of vaccine. Although within the same country of origin, people residing in different countries are exposed to different sources of information about vaccines, which could result in varied conclusions about preferred COVID-19 vaccines. A study that surveyed 34,041 participants in China found that their main sources of information came from government agencies, followed by social media, news reports of experts, and people engaging in medical work [23], whereas our study shows that Chinese Americans gained information from health officials, followed by local news, friends/family/coworker, social media, and healthcare professionals. Besides the differences found in the type of information source, information received from the same type of sources can also vary by country. This finding may apply to other immigrant populations too, such as other Asian communities and Latinx, in the U.S.

In our sample, participants accessed information regarding COVID-19 vaccines through a variety of sources. Five major information sources listed by frequency from highest to lowest include health officials and authorities, local news, family/friends/co-workers, social media, and health professionals. Increasing investments and advertisement on these resources may empower CAs’ decision-making concerning vaccination. Notably, our findings are slightly different from previous research involving 484 CAs from New York City, which indicated that print media sources were the major general health information sources, followed by the Internet [9]. The differences may indicate that CAs’ major sources of health information could change in different health topics. For instance, regarding the topic of vaccination, CAs are more likely to seek information from health officials and other authority figures. In addition, our finding indicated that participants who received higher levels of education, who were born in the U.S., and who scored higher on the AAMAS–EA scale were more likely to receive COVID-19 vaccine information from healthcare professionals. Conversely, participants with lower AAMAS–EA scores were more likely to obtain information from family/friends/co-workers. Our findings underscore the influence of immigrants’ acculturation levels on their choice of information sources regarding vaccines [7,12]. These findings suggest that future vaccine interventions or campaigns should adopt multiple dissemination channels to meet the varying preferences of immigrant audiences with diverse socioeconomic backgrounds and acculturation levels.

Interestingly, our results showed that CAs’ satisfaction with vaccination was significantly associated with self-efficacy and the subjective norm of receiving the COVID-19 vaccine. This result reinforces that increasing participants’ confidence and comfort level with receiving the vaccine may increase their satisfaction and intention to receive continuous vaccine boosters. This is especially important for those CAs who lack reliable transportation, lack adequate health insurance, have disabilities, have language barriers, and do not have easy access to the internet to make an online appointment. Additionally, most Asian cultures are predominantly collectivistic, which emphasizes the benefits of the community/society at large as opposed to focusing on the needs of individual members [24,25]. People with collectivistic values tend to engage in prosocial behavior for the best interests of society and the communities that they are members of to ensure they are following group norms [26]. Influenced by such cultural norms, CAs are likely to value and follow their perspectives, behaviors, feedback, and recommendations regarding vaccines from their community members [26]. Thus, in future vaccine campaigns or interventions, inviting community health workers, faith leaders, and social media influencers to share their experiences with vaccination via diverse information channels may be an effective communication strategy to motivate CAs to receive vaccines, especially as additional follow-up vaccinations are recommended [27].

Notably, participants’ satisfaction with the COVID-19 vaccination was significantly associated with whether they received their preferred type of vaccine when controlling other predictors. Specifically, respondents who did not receive their preferred type of COVID-19 vaccine in their initial shot were less likely to be satisfied with the process of vaccination. In our sample, 16.2% of the respondents failed to receive their preferred type of vaccine. Although multiple types of COVID-19 vaccination have been approved by the FDA, at the early stage of vaccine distribution, the public’s choice of vaccine was limited due to supply limits and cold storage requirements [28]. Because of these reasons, at the initial stage of vaccine distribution, not all products were available at each vaccination site. An experiment with 967 participants conducted in Germany in February 2021 supports that being able to choose a preferred type of vaccine enhanced participants’ intention to get vaccinated. Participants who were assigned to a non-preferred type of vaccine had lower intentions to get vaccinated [29]. Failing to accommodate individual freedom to choose a preferred type of vaccine may not only affect satisfaction with vaccination but may also breach trust in the healthcare system, which can lead to a serious barrier to achieving health equity in vaccination [28]. This finding could apply to other public health issues in addition to vaccination, such as following a recommendation to seek screening for early detection of disease or following treatment and medication recommendations. To increase the acceptance of the use of new health mandates, recommendations, treatments, and technologies, it is critical to support an individual’s free choice regarding health decisions and to provide consumers with a greater variance of available medical resources or products (e.g., vaccines, genetic testing, or biobanks).

There are several limitations in the present study. First, because we recruited a convenience sample for this study, our findings cannot be generalized to all CAs. For instance, 66.5% of our participants were female. The reason for this may be that women are more likely to respond to surveys or to be a participant in health research compared with men [30]. In addition, more than half of our participants received a high level of education and had a high income. However, these trends align with the national CA average education received (71% received some college education or above) and annual income (median annual household income: $81,600) in the U.S. [7]. Further, we did not consider measuring several relevant structural, procedural, and environmental factors that could have potentially influenced participants’ satisfaction regarding the COVID-19 vaccine in this study. Additionally, due to the nature of the cross-sectional design used in this study, causal relationship effects cannot be concluded. It is important to note that regardless of country of origin/residency, people’s acceptance, satisfaction, and preference for COVID-19 vaccines were constantly changing during different phases of the COVID-19 pandemic [31,32], suggesting that the cross-sectional design used in this study could only capture such information at one point in time, which was another limitation of this study. However, it was valuable for us to observe such differences between Chinese immigrants in the US and Chinese nationals at this critical stage when vaccine acceptance and hesitancy were dominant topics of discussion in both countries. Our study was conducted before the suspension of the Johnson & Johnson vaccine; people’s perceptions of a preferred manufacturer of vaccine may have altered after that incident. A future study may continue to track the preferred type of COVID-19 vaccine, and our study would still serve as a good reference to compare preferences at different points of the timeline.

Despite the limitations, the findings of the current study provide several relevant recommendations to guide the design and implementation of future vaccine interventions or campaigns, including adopting multiple dissemination channels to meet the varying preferences of immigrant audiences with diverse socioeconomic backgrounds and increasing satisfaction with vaccination by offering free choice and increasing self-efficacy and subjective norms of receiving vaccines. Future research should recruit study populations with more diverse socioeconomic backgrounds. We also recommend that future studies include analyses of relevant structural, procedural, and environmental factors. In addition, instead of using a single question to measure satisfaction, future studies should consider adopting a more in-depth survey instrument to measure satisfaction from multiple levels. For example, the SERVQUAL instrument is widely used in assessing health service satisfaction, which consists of five dimensions: tangibility, reliability, responsiveness, assurance, and empathy [11].

## 5. Conclusions

This study provides valuable insights into vaccine information sources, preferred vaccine types, and satisfaction levels regarding COVID-19 vaccines among CAs. Our findings indicated that health officials, local news, family/friends/co-workers, social media, and healthcare professionals were the major information sources used to learn about the COVID-19 vaccine among CAs. More than half of the participants preferred the Pfizer-BioNTech vaccine over other available types of vaccine. A majority of the participants received the type of COVID-19 vaccine they preferred, which increased their satisfaction with their vaccination experience. Overall, most participants felt satisfied with their experience with the COVID-19 vaccine. Participants with higher levels of self-efficacy and higher levels of subjective norms toward vaccination reported higher levels of satisfaction. The knowledge gained from the current study may be applied to other public health issues or future vaccine campaigns, as well as to the design of vaccine educational programs, both within and outside the CA population, with careful consideration of the use of major information sources, consumer preferences regarding varies types of vaccine, and factors influencing vaccination satisfaction that were identified in this study.

## Figures and Tables

**Table 1:** Characteristics of sample (n = 241).

Variable	N (%) or Mean (SD or Range)
Age	42.7 (16.1)
Gender	
Female	159 (66.5%)
Male	79 (33.1%)
Nonbinary	1 (0.4%)
Born in the U.S.	
No	220 (93.2%)
Yes	16 (6.8%)
Education	
High school graduate or below	20 (8.3%)
Some college or associate degree	33 (13.7%)
Four-year college degree	36 (14.9%)
Graduate school or above	152 (63.1%)
Marital status	
Married/living with a partner	192 (80.7%)
Other	46 (19.3%)
Employment	
Employed	146 (60.6%)
Other	95 (39.4%)
Religion	
Affiliated	105 (44.1%)
Unaffiliated/none	133 (55.9%)
Annual household income	
$0 to $19,999	49 (20.9%)
$20,000 to $74,999	57 (24.3%)
$75,000 or more	129 (54.9%)
Acculturation	
AAMAS–CO ^1^	5.3 (0.6)
AAMAS–EA ^2^	3.6 (1.1)
Use of the survey language	
English	138 (57.3%)
Chinese	103 (42.7%)
Failing to receive the preferred type of COVID-19 vaccine	
Yes	202 (83.8%)
No	39 (16.2%)
Self-efficacy	4.2 (2.3–5.0)
Subjective norms	4.2 (2.3–5.0)

^1^ AAMAS–CO: Asian American Multidimensional Acculturation Scale–culture of origin; ^2^ AAMAS–EA: Asian American Multidimensional Acculturation Scale–European American culture. Sum of the numbers may not equal 241 due to missing data.

**Table 2:** Predictors of satisfaction with COVID-19 vaccine among Chinese Americans (n = 241).

	Crude PR ^a^	95% CI ^b^	Adjusted PR	95% CI
Age	1.000	(0.997, 1.004)	1.003	(0.998, 1.008)
Gender				
Female	Ref		Ref	
Male	0.971	(0.863, 1.093)	0.926	(0.817, 1.050)
Nonbinary	1.179	(1.074, 1.295)	1.009	(0.749, 1.359)
Born in the U.S.				
No	Ref		Ref	
Yes	0.982	(0.770, 1.253)	0.834	(0.585, 1.188)
Education				
High school graduate or below	Ref		Ref	
Some college or associate degree	0.909	(0.731, 1.130)	0.820	(0.589, 1.140)
Four-year college degree	0.926	(0.753, 1.139)	0.871	(0.614, 1.236)
Graduate school or above	0.914	(0.775, 1.077)	0.877	(0.619, 1.243)
Marital status				
Other	Ref		Ref	
Married/living with a partner	1.097	(0.971, 1.239)	1.109	(0.959, 1.283)
Employment				
Other	Ref		Ref	
Employed	0.956	(0.853, 1.071)	0.879	(0.756, 1.022)
Religion				
Unaffiliated/none	Ref		Ref	
Affiliated	0.922	(0.817, 1.041)	0.940	(0.816, 1.083)
Annual household income	0.960	(0.896, 1.028)	0.932	(0.853, 1.020)
Acculturation				
AAMAS–CO ^1^	1.034	(0.923, 1.157)	1.113	(0.959, 1.291)
AAMAS–EA ^2^	1.002	(0.946, 1.061)	0.991	(0.917, 1.070)
Use of the survey language (Chinese vs. English)				
English	Ref		Ref	
Chinese	0.931	(0.826, 1.0494)	0.795 **	(0.681, 0.930)
Failing to receive the preferred type of COVID-19 vaccine				
No	Ref		Ref	
Yes	0.843	(0.687, 1.035)	0.810 *	(0.667, 0.984)
Self-efficacy	1.224 ***	(1.099, 1.363)	1.158 *	(1.012, 1.326)
Subjective norms	1.217 ***	(1.096, 1.352)	1.172 *	(1.010, 1.360)

^a^ Prevalence ratio; ^b^ confidence interval. * *p* < 0.05, ** *p* < 0.01, *** *p* < 0.001. ^1^ AAMAS–CO: Asian American Multidimensional Acculturation Scale–Culture of Origin; ^2^ AAMAS–EA: Asian American Multidimensional Acculturation Scale–European American culture.

## Data Availability

As a data-sharing strategy was not included in the original application for institutional-review-board review, study data are not publicly available.
